# Gastrointestinal Complications Following Ingestion of Potting Soil in a Dog

**DOI:** 10.3390/vetsci12040355

**Published:** 2025-04-10

**Authors:** Sohee Jo, Yeon Chae, Sungjae Lee, Yoonhoi Koo, Hakhyun Kim, Byeong-Teck Kang, Taesik Yun

**Affiliations:** 1 Laboratory of Veterinary Internal Medicine, College of Veterinary Medicine, Chungbuk National University, Chungbuk 28644, Republic of Korea; neco2262@naver.com (S.J.);; 2 College of Veterinary Medicine, Kyungpook National University, Daegu 41566, Republic of Korea

**Keywords:** canine, foreign body, poisoning, potting soil, veterinary toxicology

## Abstract

A 9-year-old castrated male Old English Sheepdog was referred for evaluation because of persistent vomiting, diarrhea, and anorexia following suspected ingestion of potting soil consisting of cocopeat, peat moss, perlite, zeolite, and fertilizers. Despite 3 days of symptomatic treatment at a local animal hospital, clinical signs persisted. On admission, the dog had a fever (39.7 °C) and abdominal pain. Serum biochemistry revealed elevated C-reactive protein (CRP, 210.41 mg/dL; reference interval [RI], 0–10 mg/dL) and D-dimer (371.69 ng/mL; RI, 0–250 ng/mL) levels. Abdominal radiographs showed gastric and focal duodenal dilation, while ultrasonography identified gastrointestinal dilation with fluid accumulation, hypomotility, and peritoneal edema. These findings are consistent with enteritis and peritonitis, which are likely secondary to potting soil ingestion. The dog was hospitalized and treated with intravenous fluids, antibiotics, antiemetics, gastrointestinal protectants, prokinetics, and peripheral parenteral nutrition. Significant clinical improvement was observed within 48 h, with CRP levels decreasing to 87.49 mg/dL and D-dimer to 170.48 ng/mL. This case highlights gastrointestinal complications associated with potting soil ingestion and emphasizes the importance of prevention.

## Simple Summary

Potting soil is commonly used for plant growth and is easily accessible to dogs. However, its potential gastrointestinal toxicity has not been well documented in veterinary medicine. This case report describes a 9-year-old Old English Sheepdog that developed severe vomiting, diarrhea, and anorexia following potting soil ingestion. Despite symptomatic treatment, clinical signs persisted, prompting further evaluation. Diagnostic imaging revealed gastrointestinal dilation with fluid retention and peritonitis. Intensive treatment with intravenous fluids, antibiotics, and supportive care resulted in significant improvement within 48 h. This case emphasizes the potential risks associated with potting soil ingestion in dogs and highlights the importance of preventing access to such materials.

## 1. Introduction

**Figure 1:**
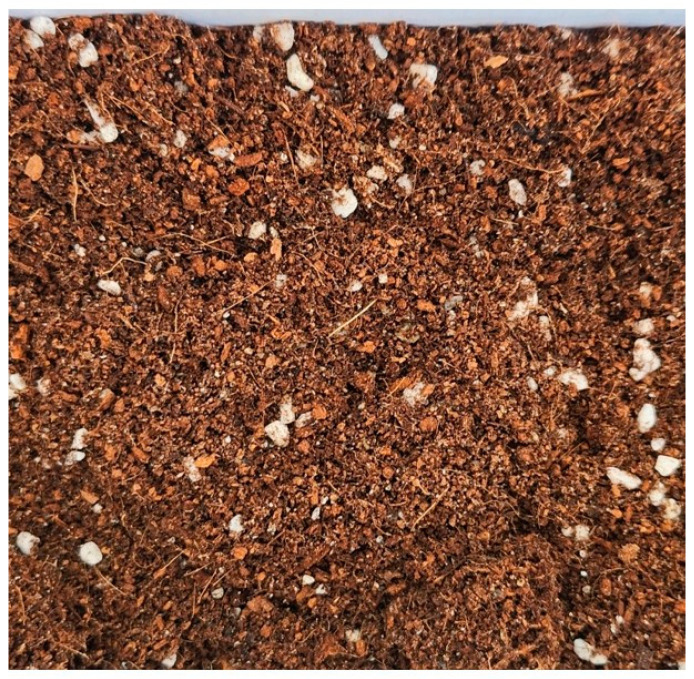
Surface appearance and texture of the potting soil used in this case.

Potting soil is a growing medium formulated to improve soil structure, moisture retention, drainage, aeration, and nutrient availability through a balanced mix of organic and inorganic components [1,2]. It optimizes root growth by enhancing anchorage, oxygen supply, and nutrient uptake. It is widely used in households and gardens and is easily accessible to dogs. Its texture and appearance resemble those of food (Figure 1), which increases the risk of ingestion, particularly in dogs that exhibit pica or exploratory behaviors [3]. As companion animals increasingly share human environments, their exposure to household materials like potting soil has become a growing concern in veterinary medicine.

Potting soil typically contains organic materials, such as coconut coir and peat moss, and inorganic components, like perlite, zeolite, and vermiculite [4]. Its composition varies depending on plant species, cultivation conditions, and manufacturer formulations. Although primarily intended for plant growth, its ingestion by dogs raises concerns regarding gastrointestinal toxicity. Some components may cause gastrointestinal disturbances through mechanical irritation, osmotic imbalance, or chemical interactions with the mucosa. While specific studies on its gastrointestinal complications in dogs are lacking, the physicochemical properties of its components suggest the potential for adverse effects. Fibrous materials such as cocopeat and peat moss may cause gastrointestinal irritation or obstruction [5,6]. Similarly, sharp and porous perlite particles can damage the intestinal mucosa [7]. The ion-exchange properties of zeolites may disrupt electrolyte balance [8,9], while chemical fertilizers containing high concentrations of nitrogen, phosphorus, and potassium can induce mucosal toxicity and inflammation [10].

This report describes the first documented case in the veterinary literature of a dog with severe gastrointestinal complications following potting soil ingestion, detailing its clinical presentation, diagnostic evaluation, and successful management. While no prior veterinary reports have described potting soil ingestion, similar complications have been reported in dogs following the ingestion of bonemeal fertilizer, underscoring the importance of recognizing soil-based materials as potential gastrointestinal hazards [11].

## 2. Case Presentation

A 9-year-old neutered male Old English Sheepdog presented with a 3-day history of vomiting, diarrhea, and anorexia. The owner directly observed the dog ingesting potting soil on a single occasion from an outdoor yard with multiple flowerpots. A significant amount of soil was noted to be missing before the onset of clinical signs, which began a few hours after ingestion. According to the owner, there were no plants known to cause gastrointestinal irritation in the garden. Throughout the clinical course, no visible potting soil particles were detected in the vomitus or diarrheic feces, although complete absence could not be confirmed due to their fine particulate nature. The ingested potting soil (Hanarum Potting Soil 50 L, Shinsung Mineral Co., Goesan-gun, Republic of Korea) is composed of cocopeat (64.3%), peat moss (15%), perlite (10%), zeolite (8%), and fertilizer (0.19%). Initial diagnostic tests for canine parvovirus, coronavirus, and heartworm disease performed at a local veterinary hospital yielded negative results. Initial treatment at a local veterinary hospital included antiemetics (maropitant, 1 mg/kg, subcutaneously, once), gastrointestinal protectants (famotidine, 1 mg/kg, orally, twice daily), and antibiotics (metronidazole, 10 mg/kg, orally, twice daily). Despite adherence to this symptomatic regimen for several days, the dog’s gastrointestinal signs failed to improve, prompting a referral to our institution.

On presentation, physical examination revealed fever (39.7 °C), 5% dehydration, and abdominal pain. Serum biochemistry demonstrated elevated levels of alkaline phosphatase (701 IU/L; reference interval [RI]: 29–97), blood urea nitrogen (61.4 mg/dL; RI: 7–25), and C-reactive protein (CRP, 210.41 mg/dL; RI: 0–10). Electrolyte imbalances included mild hyponatremia (139 mmol/L; RI: 144–151), mild hypokalemia (3.22 mmol/L; RI: 3.90–5.10), and mild hypochloremia (103 mmol/L; RI: 110–119). These abnormalities were consistent with fluid loss associated with vomiting and diarrhea, along with reduced intake.

**Figure 2:**
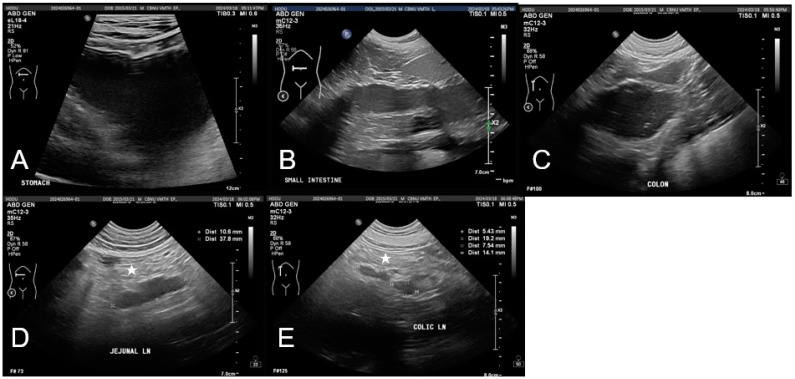
The imaging findings of gastrointestinal complications following ingestion of potting soil in a dog. (**A**) The stomach contains a significant accumulation of anechoic fluid with mild echogenic material, leading to distension. (**B**,**C**) Marked dilation of the small (**B**) and large (**C**) intestines with decreased peristalsis and fluid accumulation observed. (**D**,**E**) Jejunal lymph nodes and colic lymph nodes are mildly enlarged and appear hypoechoic. The peritoneum in the right mid-abdominal region (star) appears mildly hyperechoic and edematous.

Abdominal imaging indicated gastrointestinal dysfunction. Radiographs showed mild gastric distension and a fluid-filled small intestine without evidence of mechanical obstruction. Ultrasonography revealed gastrointestinal dilation, fluid retention, and hypomotility, suggestive of functional ileus with mild peritoneal edema and mesenteric lymphadenopathy (Figure 2). No significant abnormalities were observed in the hepatobiliary system, including liver size, echotexture, and gallbladder structure, aside from mild echogenic sludge without gallbladder wall thickening or bile duct dilation.

Fluid therapy with 0.9% sodium chloride (Normal Saline^®^, JW Pharmaceutical, Gwacheon, Republic of Korea) was initiated to correct dehydration. Potassium supplementation (KCl Injection^®^, Daihan Pharmaceutical Co., Seoul, Republic of Korea) was administered to address electrolyte imbalances. Maropitant (Cerenia^®^, Zoetis Korea, Seoul, Republic of Korea; 1 mg/kg, subcutaneously, twice daily) was given as an antiemetic, and metoclopramide (Meckool Injection^®^, Jeil Pharmaceutical Co., Seoul, Republic of Korea; 0.02 mg/kg/h) was used as a prokinetic to restore gastrointestinal motility. Metronidazole (Metrynal^®^, Daihan Pharmaceutical Co., Seoul, Republic of Korea; 10 mg/kg, intravenously, twice daily) and ampicillin-sulbactam (Sullbacin^®^, Shinpoong Pharmaceutical Co., Seoul, Republic of Korea; 20 mg/kg, intravenously, three times daily) were administered to manage potential secondary bacterial infections. Gastrointestinal protection was provided with omeprazole (Nexium Injection^®^, AstraZeneca Korea, Seoul, Republic of Korea; 1 mg/kg, intravenously, twice daily). Given the elevated D-dimer levels, dalteparin sodium (Fragmin^®^, Pfizer Korea, Seoul, Republic of Korea; 100 IU/kg, subcutaneously, three times daily) was administered as an anticoagulant to mitigate thromboembolic risk. Analgesia was managed with a fentanyl patch (Durogesic D-trans Patch^®^, Janssen Korea, Seoul, Republic of Korea; 50 µg/h). Nutritional support was maintained through peripheral parenteral nutrition using Nephrisol Inj. (Daihan Pharmaceutical Co., Seoul, Republic of Korea), calculated to meet 100% of the resting energy requirement.

The dog showed marked clinical improvement during hospitalization and was discharged five days after ingestion, following a 48 h period of intensive treatment at our hospital. Vomiting resolved, and CRP levels decreased from 210.41 to 87.49 mg/dL. In parallel, ALP levels declined from 701 to 625 IU/L, and BUN decreased markedly from 61.4 to 20.3 mg/dL following fluid therapy. At discharge, the dog showed stable vital signs and increased activity. An 8-day follow-up via telephone, without an in-person clinical examination, confirmed normal appetite, activity level, and stool consistency, suggesting sustained clinical recovery and restoration of gastrointestinal function.

## 3. Discussion

This case highlights the potential gastrointestinal and systemic complications associated with the ingestion of potting soil in dogs. The patient presented with vomiting, diarrhea, and anorexia, with laboratory findings of hyponatremia, hypokalemia, and increased CRP as well as moderate elevation in ALP and BUN levels. These findings were indicative of gastrointestinal fluid loss and systemic inflammation. The electrolyte disturbances were likely secondary to fluid depletion and reduced intake. The prompt decrease in BUN levels following fluid therapy supports the interpretation that the elevation was primarily related to prerenal azotemia due to dehydration. While the elevation in ALP may have been a non-specific finding related to inflammation or stress, it was unlikely to reflect primary hepatobiliary pathology. This interpretation is supported by the normal imaging findings and the mild improvement after fluid therapy. Diagnostic imaging revealed gastrointestinal dilation and fluid accumulation, with an edematous, hyperechoic peritoneum strongly suggesting peritonitis. The peritonitis observed in this case was presumed to be sterile, likely secondary to local inflammation caused by mucosal irritation rather than bacterial contamination due to gastrointestinal perforation. Given the range of mechanical and chemical interactions that may occur in the gastrointestinal tract after soil ingestion, it is important to consider not only the presence of systemic signs but also their underlying pathophysiological basis. A thorough assessment of potting soil components is essential to determine their pathological impact and guide appropriate treatment strategies.

Cocopeat, a fibrous material derived from coconut husk, is widely used in horticulture because of its moisture retention and aeration properties, absorbing up to 8–10 times its weight in water [5]. While beneficial for plant growth, its ingestion may interfere with normal gastrointestinal function by accumulating in the digestive tract and causing mechanical irritation of the gastrointestinal mucosa. When consumed in large amounts, its high fiber content may lead to inflammation, vomiting, and diarrhea. Additionally, its small particle size (0.425–4 mm) and high insoluble fiber content may further contribute to mucosal irritation and altered gastrointestinal transit times [12,13]. It has a neutral to slightly acidic pH (5.2–6.8), but this level decreases over time, indicating that it becomes increasingly acidic and lacks buffering capacity in the low-pH gastric conditions. [5,13]. These properties may make cocopeat particularly problematic when ingested in large quantities, especially in patients with pre-existing gastrointestinal sensitivity. These findings highlight the need for further research on the gastrointestinal effects of cocopeat ingestion in dogs.

Peat moss, a decomposed plant material, is widely used owing to its ability to enhance soil moisture retention, retaining up to 20 times its weight in water. Although beneficial for horticulture, its ingestion may cause irritation of the gastrointestinal mucosa due to its acidic pH (3.8–4.3). This can disrupt the protective mucus layer and exacerbate conditions such as gastritis or ulceration [6]. Additionally, certain organic acids present in peat, including humic and fulvic acids, have been reported to modulate gastrointestinal motility, potentially leading to altered transit times [14,15]. However, their specific effects in canine species remain unclear. Peat is also known for its adsorptive properties, which allow it to bind to toxins and heavy metals; however, excessive accumulation in the gastrointestinal tract may interfere with normal digestion and absorption [6]. Therefore, both its chemical properties and adsorptive behavior warrant closer scrutiny in the context of companion animal exposure.

Furthermore, peat can harbor environmental mycobacteria, including the *Mycobacterium avium* complex, which has been associated with granulomatous infections in pigs [16]. Studies have reported that pigs exposed to peat developed tuberculous lesions in their lymph nodes, raising concerns about potential contamination risks. Although the clinical relevance of such contamination in dogs has not been established, its potential effect on immunocompromised individuals warrants further investigation.

Perlite is an expanded volcanic glass commonly used in horticulture to enhance soil aeration and drainage. Heated to 900–1000 °C, volcanic rock expands up to 13 times its original volume, forming a lightweight, porous structure that enhances soil properties [7,17]. Perlite particles typically range from 1.4 mm to 4 mm in diameter, with a rough, irregular surface that may mechanically irritate the gastrointestinal mucosa upon ingestion [12]. As an indigestible material, perlite may accumulate within the gastrointestinal tract, posing a risk of mechanical obstruction, particularly in smaller dogs with narrower intestinal lumen [18]. Additionally, its unique physical properties, including its ability to retain moisture on its surface rather than absorb it internally, suggest that it may alter the gastrointestinal transit time or fecal consistency when ingested in significant amounts [7]. Furthermore, perlite particles have been observed to aggregate under certain conditions, raising concerns about their potential to form compact masses in the digestive tract. This potential for aggregation and obstruction further underscores the importance of evaluating ingested soil materials not only for chemical content but also for physical form.

Zeolite is a naturally occurring aluminosilicate mineral widely recognized for its cation-exchange capacity and porous structure. It is commonly used in soil conditioning because of its ability to absorb and release essential ions, such as ammonium, potassium, and nitrate, thereby enhancing soil fertility and water retention [9]. However, when ingested, its strong ion-exchange capacity may interfere with electrolyte balance by binding potassium and sodium, potentially leading to hypokalemia and hyponatremia [8]. Additionally, its non-digestible, crystalline structure with rough edges can mechanically irritate the gastrointestinal mucosa, causing localized inflammation or ulceration. Zeolite particles typically range from 2 to 5 mm in diameter. Their shape and texture may increase the risk of mechanical obstruction, particularly in smaller dogs [8]. Furthermore, its high affinity for heavy metals raises concerns about its potential to act as a carrier of toxic contaminants if sourced from polluted environments [9]. These considerations highlight the importance of monitoring the source and purity of zeolite used in commercially available potting mixes.

Fertilizers contain essential macronutrients, including nitrogen, phosphorus, and potassium, which support plant growth by promoting leaf and stem development, root expansion, and cellular water balance [19,20]. However, fertilizer ingestion can irritate the gastrointestinal mucosa through changes in the gastric pH and osmotic imbalance, potentially leading to vomiting, hypersalivation, and diarrhea [10]. Nitrogen-based fertilizers may react with gastric fluid to form ammonia, which can cause protein denaturation and mucosal injury. At the same time, calcium phosphate can dissolve in gastric acid, further aggravating mucosal irritation [10]. High phosphorus and potassium concentrations in fertilizers may increase the luminal osmotic pressure, leading to osmotic diarrhea and fluid shifts that contribute to dehydration. Electrolyte abnormalities such as hyperkalemia and acidosis have been reported in humans after ingestion of NPK liquid fertilizers, especially in large amounts. In this case, hypokalemia was observed, which may reflect fluid loss and other contributing factors rather than direct potassium toxicity [21]. The fertilizer concentration in this potting soil was relatively low (0.19%), making significant toxicity unlikely. Based on this concentration and the patient’s body weight (40 kg), approximately 10.5 kg of potting soil would need to be ingested to reach the threshold dose (0.5 g/kg) for potential gastrointestinal toxicity [22]. Thus, clinically relevant toxicity from fertilizer components appears improbable in this case. While large-volume ingestion of concentrated fertilizers has been associated with acute kidney injury and gastrointestinal hemorrhage [10], the minimal exposure in this case suggests that the fertilizer components were not the primary cause of the clinical signs. Nonetheless, cumulative exposure over time in cases of repeated ingestion cannot be fully dismissed, especially in dogs with existing gastrointestinal sensitivities.

Despite these findings, significant knowledge gaps remain regarding the dose-dependent toxicity, absorption kinetics, and long-term systemic effects of potting soil components in dogs [23]. This case also highlights one of these limitations, as the ingested volume could not be quantified and was based solely on owner observation, making it difficult to establish a clear dose–response relationship. To address these limitations, future studies should include standardized dose–response experiments to identify the threshold at which gastrointestinal disturbances occur following ingestion of components such as cocopeat and zeolite. In addition, histopathological evaluations of gastrointestinal tissues may help assess mucosal irritation or inflammation associated with such exposures. Such studies would provide valuable insights into not only the local effects on the gastrointestinal tract but also the potential systemic consequences of chronic or repeated low-dose exposure. Addressing these knowledge gaps will refine veterinary diagnostic protocols and treatment strategies, ultimately improving clinical outcomes. Furthermore, increasing the awareness of pet owners and implementing pet-safe gardening practices, along with restricting access to hazardous materials, are essential steps for reducing exposure risks and improving animal health.

## 4. Conclusions

Potting soil ingestion can cause gastrointestinal complications in dogs, especially in those with pica, due to its physical properties. Specifically, the water-absorptive properties of some potting soil components may have contributed to the development of diarrhea. This case underscores the need to consider potting soil ingestion as a differential diagnosis in dogs with acute gastrointestinal symptoms, particularly in environments where gardening materials are easily accessible. Preventive measures such as restricting access to potting soil and promoting pet-safe gardening practices may help reduce these cases.

## Data Availability

The data presented in this study are available in the manuscript.
